# Alzheimer’s Spinal Pathology: Neuronal, Glial, and Cholesterol Metabolic Changes in Female and Male 5xFAD Mice

**DOI:** 10.3390/ijms27083593

**Published:** 2026-04-17

**Authors:** Xiaochuan Wang, William Harnett, Xinhua Shu, Hui-Rong Jiang

**Affiliations:** 1 Strathclyde Institute of Pharmacy and Biomedical Science, University of Strathclyde, 161 Cathedral Street, Glasgow G4 0RE, UK; xiaochuan.wang.2016@uni.strath.ac.uk (X.W.); w.harnett@strath.ac.uk (W.H.); 2 Department of Biological and Biomedical Sciences, Glasgow Caledonian University, Glasgow G4 0BA, UK

**Keywords:** Alzheimer’s disease, 5xFAD mice, spinal cord, β-amyloid, neuroinflammation, astrocyte, cholesterol metabolism

## Abstract

Alzheimer’s disease (AD) is the most prevalent form of dementia and is characterized by abnormal aggregation of β-amyloid (Aβ) peptides, tau proteins, and neuroinflammation in the central nervous system (CNS). While most AD research has focused on the brain, the molecular pathology of the spinal cord remains poorly understood. In this study, we investigated amyloid pathology, neurodegeneration, neuroinflammation, and cholesterol metabolism across distinct regions of the spinal cord and examined sex-specific differences using a model of AD, 5xFAD mice. Our data reveal that Aβ accumulation was restricted to the cervical spinal cord at 3 months but was evident in all areas of the spinal cord by 9 months, with similar patterns in both female and male animals. Despite this early and progressive Aβ deposition, no significant neuronal loss was observed in the ventral horn of the cervical spinal cord in either sex at 3 or 9 months of age. In contrast, there was a significant positive correlation between Aβ deposition and Iba1+ cell density in the spinal cord of 5xFAD mice. The number of Iba1+ cells in both the grey and white matter was significantly increased in female and male 5xFAD mice compared with age-matched wild-type (WT) littermates at 9 months of age. Astrocytic responses, however, were sex-specific: female, but not male, 5xFAD mice exhibited a significant increase in GFAP+ astrocytes in the grey matter of the thoracic and lumber spinal cord at 9 months compared with 3 months and relative to age-matched WT controls in the cervical and thoracic spinal cord. Furthermore, GFAP+ area in the thoracic spinal cord was significantly higher in female 9-month-old 5xFAD mice compared with their male counterparts, indicating a female-specific astrocytic response in AD spinal cord pathology. Our data also show an increase in free cholesterol (Filipin+ area) in 5xFAD mice at 9 months relative to WT controls, accompanied by altered expression of cholesterol metabolism genes, including downregulation of *Abca1*, *Cyp46a1* and *Cyp27a1*. Collectively, these findings provide new insights into AD progression in the spinal cord, highlighting molecular pathology of AD extending beyond the brain.

## 1. Introduction

Alzheimer’s disease (AD) is currently the leading cause of dementia worldwide and poses a major global health challenge [1]. AD is primarily characterized by the abnormal accumulation of amyloid-β (Aβ) peptides and hyperphosphorylated tau protein within the central nervous system (CNS), ultimately leading to widespread neurodegeneration. While therapeutic strategies targeting Aβ peptides have dominated clinical research, their limited success underscores the need to broaden our understanding of additional pathological mechanisms [2]. Increasing evidence highlights the substantial involvement of neuroinflammation [3] and cholesterol-metabolism dysregulation [4] in AD pathogenesis, suggesting that these processes may represent important therapeutic targets.

Glial cells in the CNS, including microglia, astrocytes and oligodendrocytes, are essential for maintaining homeostasis and coordinating immune responses [3,5,6]. In AD, these cells become highly reactive and actively contribute to disease pathophysiology. Microglia and astrocytes are recruited to sites of Aβ plaque deposition and neurofibrillary tangle formation, where they attempt to clear these pathogenic aggregates through phagocytosis and degradation [7,8]. However, chronic activation of glial cells can lead to the sustained release of proinflammatory cytokines, chemokines, and reactive oxygen species, contributing to a deleterious neuroinflammatory environment. Glial cells also play a role in activating the complement system and further promoting the progression of neurodegeneration [9]. While proper cholesterol clearance is essential for maintaining CNS homeostasis, disruption of lipid homeostasis is another pathological hallmark increasingly recognized in AD [10]. Genetic studies have identified several key risk genes in AD that are widely recognized for their roles in lipid metabolism, including APOE, APOJ, ABCA1, and ABCA7 [11]. In addition, a number of lipid metabolism-related genes, including LXR and ApoER2, have been implicated in the regulation of amyloid precursor protein (APP) metabolism [12]. Dysregulation of lipid pathways has been shown to contribute to multiple pathogenic processes in AD, including amyloid accumulation, chronic neuroinflammation, and blood–brain barrier dysfunction, underscoring the complex interplay between lipid metabolism and neurodegeneration [13].

Neuropathology in AD not only leads to cognitive decline, a hallmark clinical feature, but also causes motor impairments that often emerge at early disease stages and progressively worsen over time [14]. However, most AD research has focused on cognitive-associated brain regions, such as the hippocampus, while motor-related regions—including the motor cortex and spinal cord—remain comparatively understudied. The spinal cord plays a central role in sensorimotor processing and motor output, and characterizing its pathological alterations may provide new insights into the mechanisms underlying AD-associated motor deficits.

In this study, we investigated the pathology of the spinal cord in a 5xFAD mouse model of AD, with a specific focus on Aβ deposition, neuronal loss, neuroinflammation, and alterations in cholesterol metabolism. We further examined responses among neurons, astrocytes, microglia, and oligodendrocytes across different spinal cord regions and assessed whether these pathological changes differ between female and male animals. By exploring AD-related pathology within the spinal cord, this work aims to expand current understanding of motor-associated neurodegeneration and identify potential mechanisms linking neuroinflammation and cholesterol metabolism to spinal cord dysfunction in AD.

## 2. Results

### 2.1. Accumulation of Aβ Plaques in the Spinal Cord of 5xFAD Mice and Controls

**Figure 1:**
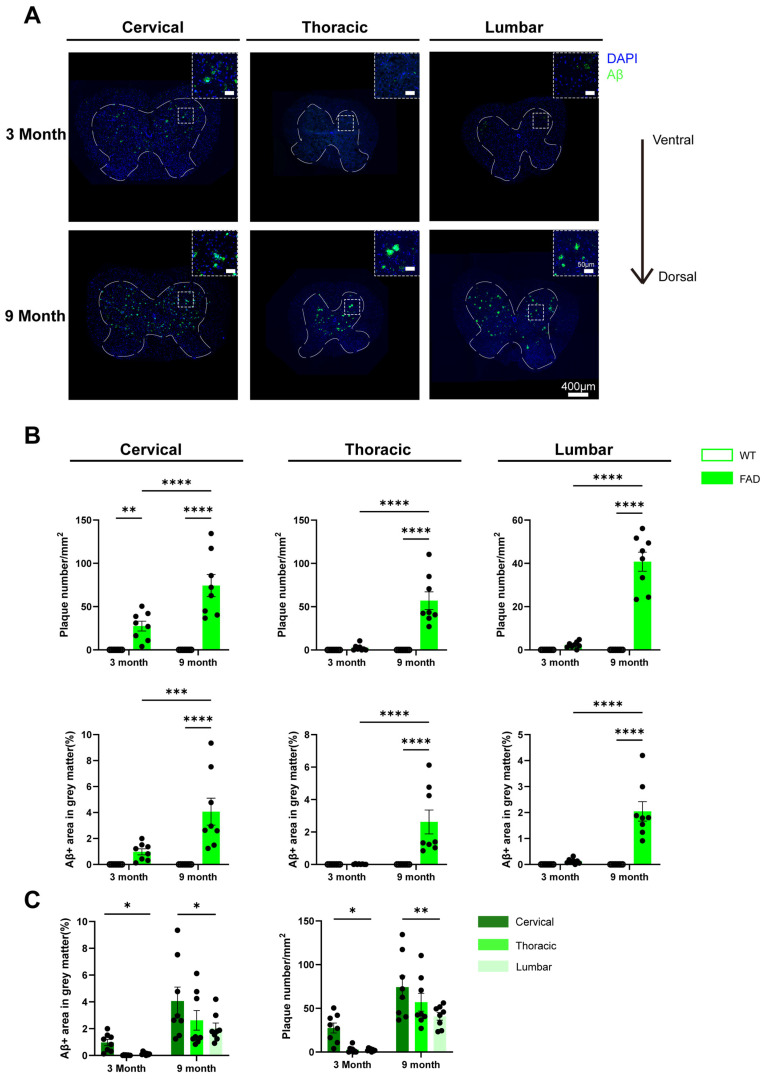
Presence of Aβ plaques in the spinal cord of 5xFAD mice. Accumulation of plaques in cervical, thoracic, and lumbar spinal cord of 5xFAD mice and WT littermates was assessed by immunostaining with anti-Aβ antibodies. (**A**) Representative images of Aβ staining in cervical, thoracic and lumbar spinal cord of 3-month (3M) and 9-month-old (9M) WT and 5xFAD mice. Area within the white dashed line was recognized as grey matter. Close-up figure (top right corner of each image) of ventral laminae is shown, with size bar equal to 50 μm. (**B**) Quantification of the percentage of Aβ-covered area and plaque number in the grey matter of the cervical, thoracic and lumbar spinal cord tissues of 3M and 9M WT and 5xFAD mice. (**C**) Regional comparison of the percentage of Aβ-covered area (left panel) and plaque number (right panel) across cervical, thoracic, and lumber spinal cord segments in 5xFAD mice at 3M and 9M of age. Data are presented as the mean ± SEM (*n* = 8 mice per group; mixed sexes). *: *p* < 0.05, **: *p* < 0.01, ***: *p* < 0.001, and ****: *p* < 0.0001.

To assess amyloid pathology in different regions of the spinal cord, slices from the cervical, thoracic, and lumbar regions of 5xFAD mice and wild-type (WT) littermates at 3 and 9 months of age were stained with an anti-Aβ antibody. As shown in Figure 1A, Aβ plaques were first detected in the cervical region of 3-month-old 5xFAD mice but were essentially absent in the thoracic and lumbar regions. However, by 9 months of age, Aβ plaques were evident in all three regions, predominantly in the ventral region of the grey matter.

As amyloid plaques were predominantly localized to the grey matter, we quantified both the percentage of grey matter area covered by Aβ-positive staining and the number of plaques larger than 100 µm^2^ to assess plaque burden (Figure 1B). At 3 months of age, 5xFAD mice exhibited significantly more plaques in the cervical spinal cord compared with WT mice. By 9 months, both Aβ coverage and plaque number were markedly increased across all spinal cord regions in 5xFAD mice relative to WT controls. Moreover, within 5xFAD mice, Aβ coverage and plaque number were significantly higher in the cervical, thoracic, and lumbar regions of 9-month-old mice compared with 3-month-old mice. Regional comparisons indicate that Aβ coverage and plaque numbers were significantly higher in the cervical tissues than in the lumber regions of 5xFAD mice at 3 and 9 months of age (Figure 1C). Two-way ANOVA revealed significant effects of age and genotype on both Aβ coverage and plaque number across regions. In addition, significant age × genotype interactions were observed for plaque number in all regions and for Aβ coverage in the lumbar region. The findings indicate that the effect of genotype on Aβ accumulation and plaque deposition is age-dependent and becomes more pronounced in older animals.

### 2.2. No Neuronal Loss in Spinal Cord of 5xFAD Mice

Next, we investigated whether neuronal loss occurs in regions of the spinal cord exhibiting Aβ plaque deposition. Neurons were visualized and quantified using immunohistochemistry with an anti-NeuN antibody. Neuronal morphology differs between the ventral and dorsal regions of the spinal cord, with ventral neurons exhibiting larger cell bodies compared to dorsal neurons. Consistent with previous studies [15], these differences reflect distinct neuronal populations in the dorsal and ventral spinal cord. Notably, few Aβ plaques were detected in the dorsal lamina, and thus neuronal quantification was performed specifically in the ventral regions (laminae V–VIII).

**Figure 2:**
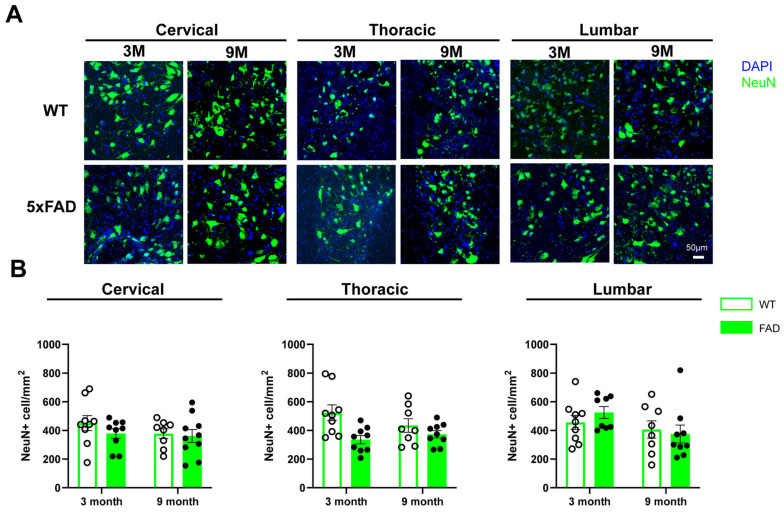
NeuN+ neuronal density in the spinal cord tissues of WT and 5xFAD mice. Cervical, thoracic and lumbar regions of spinal cord tissues were collected from WT and 5xFAD mice and processed for immunohistochemical staining with an antibody against NeuN. (**A**) Representative images of NeuN staining in the ventral horn of cervical, thoracic and lumbar spinal cord from 3-month (3M) and 9-month-old (9M) WT and 5xFAD mice. (**B**) Quantification of NeuN+ neuronal density in the ventral horn of cervical, thoracic and lumbar spinal cord of 3M- and 9M-old mice. Data are presented as the mean ± SEM (*n* = 8–9 mice per group; mixed sexes). No statistically significant differences were observed among groups.

In the ventral cervical spinal cord, no significant differences in the number of NeuN+ neurons were observed between 3-month-old WT and 5xFAD mice, nor in the ventral thoracic or lumbar regions (Figure 2A). At 9 months of age, the total number of NeuN+ neurons remained comparable between WT and 5xFAD mice across all spinal cord segments (Figure 2B). These findings suggest that, despite the presence of Aβ plaques, there is no significant loss of neuron cells in the spinal cord of 5xFAD mice up to 9 months of age.

### 2.3. Region- and Age-Dependent Microglial Activation in 5xFAD Spinal Cord

To investigate whether amyloidosis drives glial recruitment and activation, we examined microglia, astrocytes, and oligodendrocytes in the spinal cord of 5xFAD mice using immunohistochemistry for Iba1, GFAP, and Olig2, respectively.

**Figure 3:**
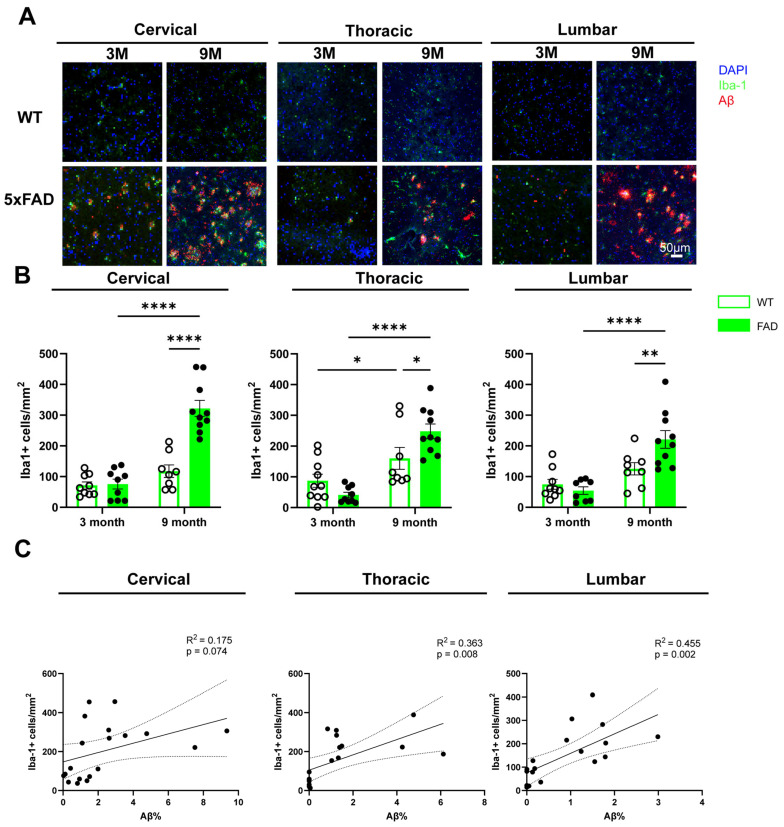
The number of Iba+ microglia in the spinal cord tissues of WT and 5xFAD mice. Cervical, thoracic and lumbar spinal cord tissues were collected from WT and 5xFAD mice and processed for immunohistochemical staining with antibodies against Aβ and Iba1. (**A**) Representative images of Iba1 and Aβ staining in the grey matter of cervical, thoracic and lumbar spinal cord of 3-month (3M) and 9-month-old (9M) WT and 5xFAD mice. (**B**) Quantification of Iba1+ microglial cell density in the grey matter of the cervical, thoracic and lumbar spinal cord of 3-month and 9-month-old WT and 5xFAD mice. Data are presented as the mean ± SEM (*n* = 8–10 mice per group; mixed sexes). (**C**) Linear regression analysis showing the correlation between percentage of Aβ-covered area and Iba1+ microglia density in the grey matter of cervical, thoracic and lumbar spinal cord of 3-month and 9-month-old 5xFAD mice (combined *n* = 18 mice; mixed sexes). *: *p* < 0.05, **: *p* < 0.01 and ****: *p* < 0.0001.

Microglia are resident CNS myeloid cells that act as local macrophages, regulating neuronal and synaptic function and clearing dead cells and misfolded proteins, including Aβ [16]. Iba1, a marker upregulated during microglial activation, was used to assess microglial response to Aβ plaque development. Iba1+ cells were observed in the cervical, thoracic, and lumbar spinal cord of both WT and 5xFAD mice and were frequently colocalised with Aβ deposits (Figure 3A).

Quantification of Iba1+ cells per mm^2^ in the grey matter revealed no significant differences between WT and 5xFAD mice at 3 months of age. At 9 months, 5xFAD mice exhibited a significantly higher microglial density in the cervical, thoracic and lumbar spinal cord compared with WT littermates. In addition, Iba1+ cell density was significantly increased across all spinal cord regions in the 9-month-old 5xFAD mice relative to the 3-month-old mice (Figure 3B). A significant age-related (9M vs. 3M) increase in thoracic Iba1+ cell density was also observed in WT mice, whereas no such changes were detected in the cervical or lumbar regions. Two-way ANOVA revealed a significant interaction between age and genotype in the cervical (F(1,32) = 25.39, *p* < 0.0001), thoracic (F(1,33) = 8.137, *p* = 0.007), and lumbar (F(1,31) = 7.308, *p* = 0.011) grey matter, indicating that the effect of genotype on microglial density depends on age across spinal cord regions. In addition, significant main effects of age were observed in all regions (cervical: F(1,32) = 54.42, *p* < 0.0001; thoracic: F(1,33) = 35.43, *p* < 0.0001; lumbar: F(1,31) = 25.75, *p* < 0.0001). These findings suggest that age is a main driver of increased Iba1 expression, while the effect of genotype is age dependent.

Having observed co-localisation of Aβ and Iba1 in the tissues and the significant increase in Iba1+ cells in 9-month-old 5xFAD mice, we next examined the relationship between Aβ burden and Iba1 expression. In the thoracic and lumbar spinal cord of 9-month-old 5xFAD mice, a significant positive correlation was identified between Aβ deposition and the density of Iba1+ microglial cells (Figure 3C).

### 2.4. Astrocytic Reactivity Surrounding Aβ Plaques in 5xFAD Spinal Cord

**Figure 4:**
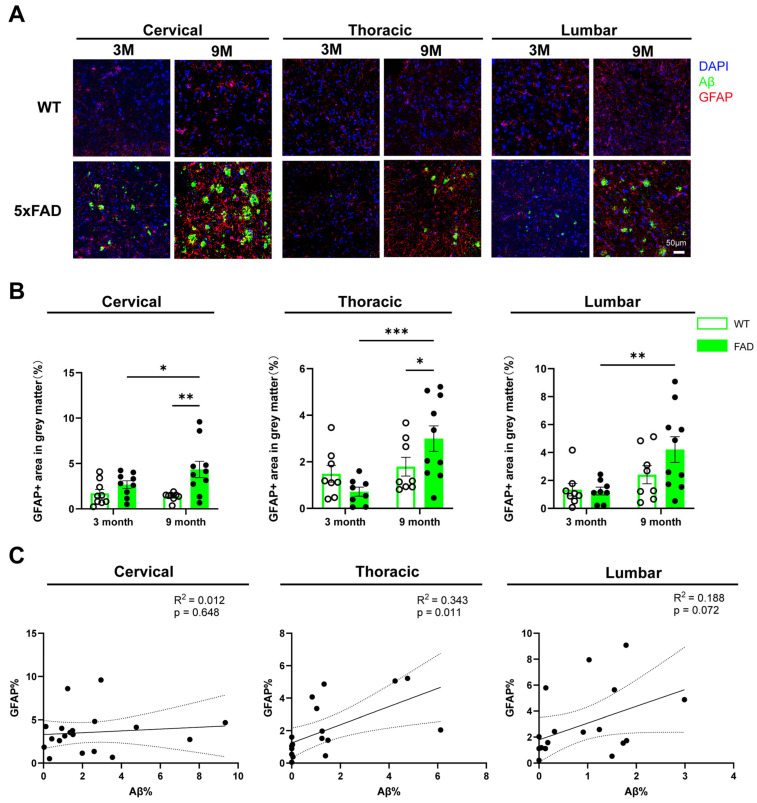
Astrocyte responses in the spinal cord of WT and 5xFAD mice. Cervical, thoracic and lumbar regions of spinal cord tissues were collected from WT and 5xFAD mice and processed for immunohistochemical staining with antibodies against Aβ and GFAP. (**A**) Representative images of GFAP staining in the grey matter of cervical, thoracic and lumbar spinal cord of 3-month (3M) and 9-month-old (9M) WT and 5xFAD mice. (**B**) Quantification of GFAP+ area (%) in the grey matter of cervical, thoracic and lumbar spinal cord tissues from of 3-month and 9-month-old WT and 5xFAD mice. Data are presented as the mean ± SEM (*n* = 8–10 mice per group; mixed sexes). (**C**) Linear regression analysis showing the correlation between the percentage of Aβ-covered area and GFAP+ area in the grey matter of cervical, thoracic and lumbar spinal cord of 3-month and 9-month-old 5xFAD mice (combined *n* = 18 mice; mixed sexes). *: *p* < 0.05, **: *p* < 0.01, and ***: *p* < 0.001.

Astrocytic responses to Aβ were assessed using GFAP immunostaining, and the percentage of GFAP+ area in the grey matter was quantified. In 5xFAD mice, astrocytes were frequently localized around Aβ plaques within the grey matter (Figure 4A). At 3 months of age, no significant differences in GFAP coverage were observed between WT and 5xFAD mice. By 9 months, however, 5xFAD mice displayed a significantly increased percentage of GFAP+ area in the cervical and thoracic spinal cord compared with age-matched WT controls. In 5xFAD mice, GFAP coverage was significantly increased across all spinal cord regions in 9-month-old 5xFAD mice compared with those at 3 months, whereas WT mice showed no significant changes over this period (Figure 4B). In the cervical spinal cord, two-way ANOVA showed a significant effect of genotype only (F(1,32) = 11.05, *p* = 0.002). In the thoracic region, significant effects were observed for age (F(1,30) = 9.243, *p* = 0.005) and the age × genotype interaction (F(1,30) = 5.422, *p* = 0.027). In the lumbar spinal cord, a significant effect of age was detected (F(1,30) = 9.076, *p* = 0.005), but neither genotype nor the interaction reached statistical significance.

We further examined the relationship between Aβ burden and astrocytic activity. A significant positive correlation was observed between the percentage of Aβ-covered area and GFAP+ region in the thoracic spinal cord (Figure 4C). These findings indicate region-specific and partially age-dependent changes in GFAP coverage, with effects varying across spinal cord levels.

### 2.5. Oligodendrocyte Density Remains Largely Unchanged in the Spinal Cord of 5xFAD Mice

**Figure 5:**
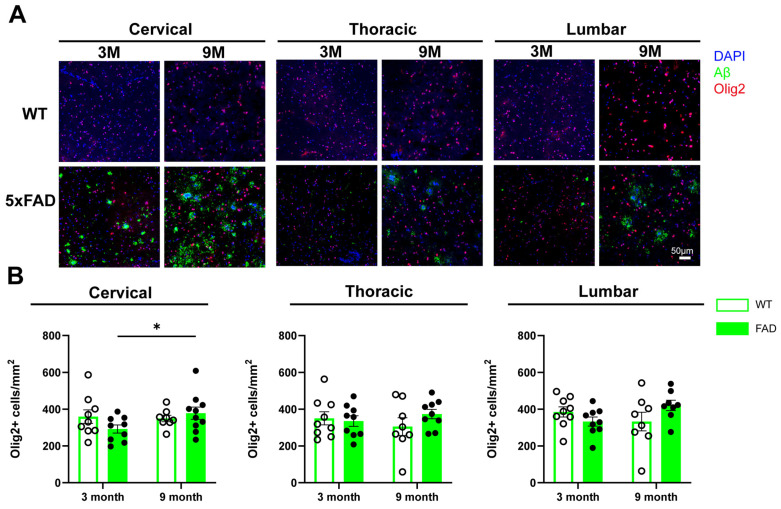
Distribution of oligodendrocytes in the spinal cord tissues of WT and 5xFAD mice. Cervical, thoracic and lumbar spinal cord tissues were collected from WT and 5xFAD mice and processed for immunohistochemical staining with antibodies against Aβ and Olig2. (**A**) Representative images of Olig2 and Aβ staining in the grey matter of cervical, thoracic and lumbar spinal cord from 3-month and 9-month-old WT and 5xFAD mice. (**B**) Quantification of Olig2+ cell density in the grey matter of the cervical, thoracic and lumbar spinal cord of 3-month and 9-month-old WT and 5xFAD mice. Data are presented as the mean ± SEM (*n* = 8–10 mice per group; mixed sexes). *: *p* < 0.05.

Oligodendrocytes, the most abundant glial cell type in the human CNS [17], are primarily responsible for myelination and can contribute to neuroinflammation through the release of immune cytokines and chemokines, as well as the upregulation of complement-related genes [18,19]. To evaluate oligodendrocyte responses to Aβ deposition and aging, Olig2, a pan-oligodendrocyte marker, was used to assess oligodendrocyte distribution and density in the spinal cord. Our data show that Olig2+ cells were widely distributed throughout both the grey and white matter of the spinal cord in WT and 5xFAD mice (Figure 5A). No significant differences in Olig2+ cell density were observed between WT and 5xFAD mice at either 3 or 9 months of age. However, in the 5xFAD mice, Olig2+ cell density in cervical grey matter was significantly increased at 9 months compared with 3-months of age (Figure 5B).

### 2.6. Altered Cholesterol Metabolism in the Spinal Cord of 5xFAD Mice

**Figure 6:**
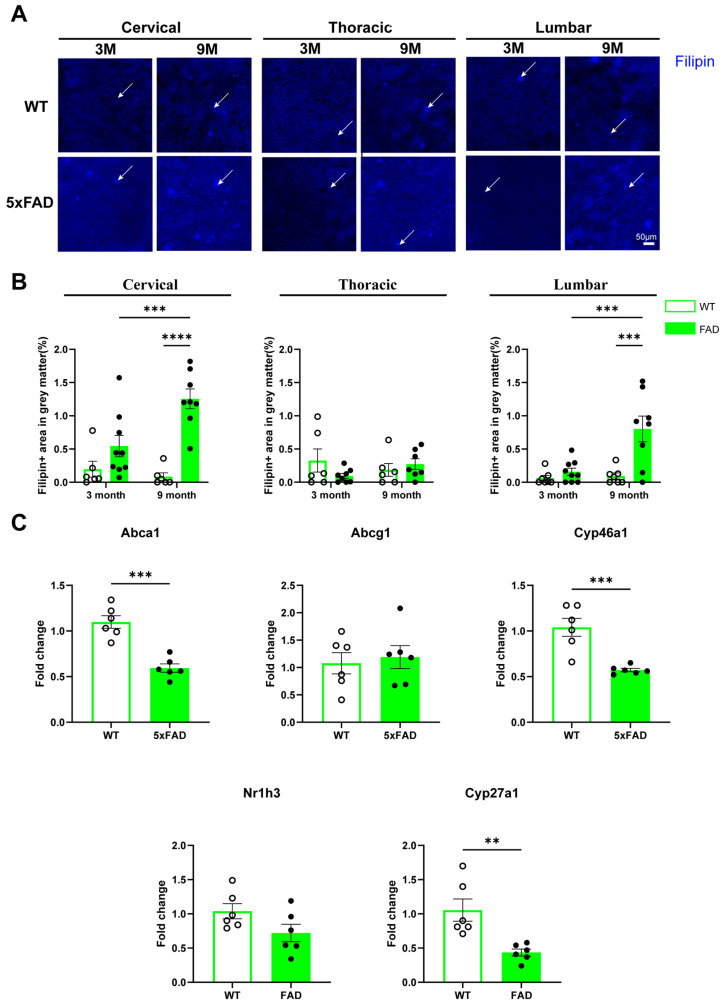
Free cholesterol levels in the spinal cord of WT and 5xFAD mice. Cervical, thoracic and lumbar spinal cord tissues were collected from WT and 5xFAD mice and subjected to Filipin staining to detect free cholesterol. (**A**) Representative images of Filipin staining in the grey matter of the cervical, thoracic and lumbar spinal cord of 3-month (3M) and 9-month-old (9M) WT and 5xFAD mice. White arrows indicate examples of positive Filipin staining. (**B**) Quantification of Filipin+ in the grey matter of the cervical, thoracic and lumbar spinal cord area of 3-month and 9-month-old WT and 5xFAD mice. Data are presented as the mean ± SEM (*n* = 8–10 mice per group; mixed sexes). (**C**) Relative mRNA expression levels of cholesterol metabolism-related genes, *Abca1*, *Abcg1*, *Cyp46a1*, *Nr1h3*, and *Cyp27a1*, in the spinal cord of 9-month-old WT and 5xFAD mice (*n* = 6 mice per group; mixed sexes). **: *p* < 0.01, ***: *p* < 0.001, and ****: *p* < 0.0001.

To determine whether cholesterol metabolism is altered in the spinal cord during AD development, Filipin staining was performed to assess free cholesterol levels in spinal cord tissues from 5xFAD mice and WT littermates. As shown in Figure 6A,B, the area of Filipin+ staining was significantly increased in the cervical and lumbar spinal cord of 9-month-old 5xFAD mice compared with both age-matched WT controls and 3-month-old 5xFAD mice. In the grey matter of cervical and lumbar regions, two-way ANOVA revealed significant effects of age, genotype, and their interaction, supporting an age- and genotype-dependent increase in free cholesterol accumulation. In contrast, no significant effects of age, genotype, or their interaction were detected in the thoracic spinal cord. These results indicate an age- and region-dependent accumulation of free cholesterol in the spinal cord associated with AD pathology.

To further evaluate disruption in cholesterol homeostasis, the expression of key genes involved in cholesterol synthesis, transport, and metabolism was analysed in the cervical spinal cord of 9-month-old 5xFAD and WT mice. As shown in Figure 6C, expression levels of *Abca1*, *Cyp46a1*, and *Cyp27a1* were significantly reduced in 5xFAD mice compared to WT controls. In comparison, no significant differences were observed in the expression of *Nr1h3* (Lxrα) and *Abcg1* between the two groups.

### 2.7. White Matter Microglial and Lipid Abnormalities in the Spinal Cord of 5xFAD Mice

**Figure 7:**
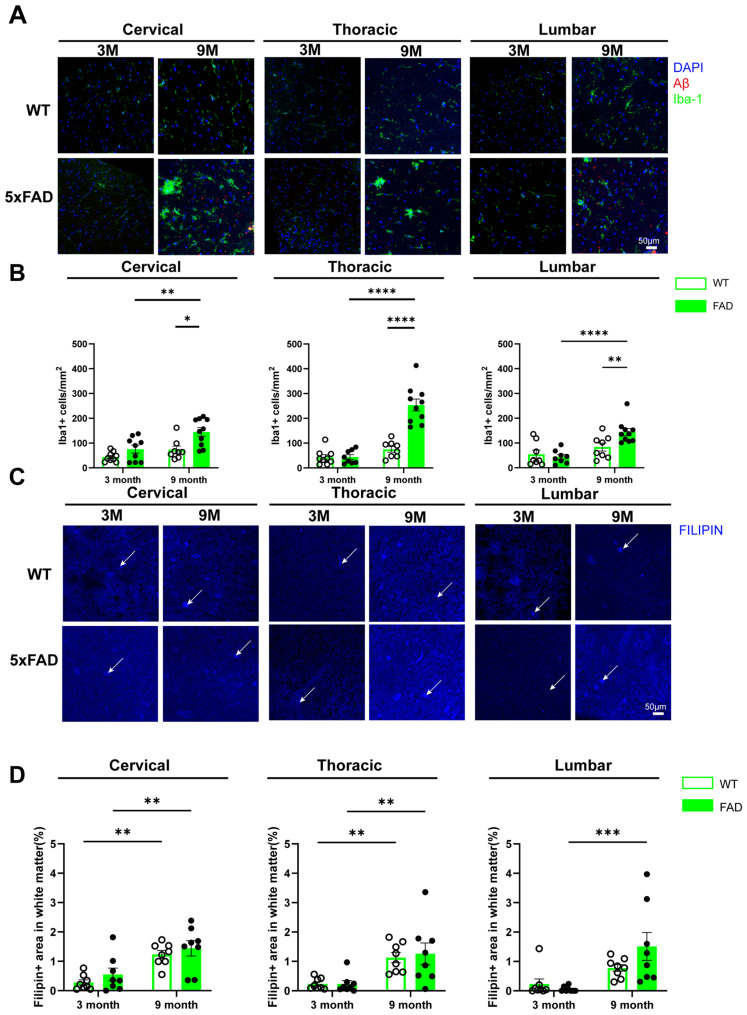
White matter microglial and lipid dysregulations in the spinal cord of 5xFAD mice. (**A**,**C**) Representative images of Iba1 (**A**) and Filipin (**C**) staining in the white matter of the cervical, thoracic and lumbar spinal cord from 3-month (3M) and 9-month-old (9M) WT and 5xFAD mice. White arrows indicate examples of positive Filipin staining. (**B**,**D**) Quantification of Iba1+ cells (**B**) and percentage of Filipin+ area (**D**) in the white matter of the cervical, thoracic and lumbar spinal cord of 3-month and 9-month-old WT and 5xFAD mice. Data are presented as the mean ± SEM (*n* = 6–10 mice per group; mixed sexes). *: *p* < 0.05, **: *p* < 0.01, ***: *p* < 0.001, and ****: *p* < 0.0001.

Previous research has identified a distinct population of white matter-associated microglia characterized by enhanced phagocytic activity and altered lipid metabolism in aging and neurodegeneration [20]. Building on these findings, we investigated microglial activation and lipid accumulation within the spinal cord white matter. We found a significant increase in the number of Iba1+ microglial cells in the white matter of 9-month-old 5xFAD mice compared with age-matched WT littermates or 3-month-old 5xFAD mice (Figure 7A,B), indicating an age-dependent enhancement in neuroinflammatory responses in this AD model. In the cervical white matter, two-way ANOVA showed significant main effects of age (F(1,32) = 11.57, *p* = 0.002) and genotype (F(1,32) = 12.74, *p* = 0.001), with no significant interaction, indicating independent contributions of aging and genotype to microglial accumulation. In the thoracic white matter, strong effects were observed for age (F(1,31) = 53.65, *p* < 0.0001), genotype (F(1,31) = 29.60, *p* < 0.0001), and their interaction (F(1,31) = 29.12, *p* < 0.0001), suggesting a synergistic effect of aging and amyloid pathology in this region. In the lumbar white matter, age (F(1,30) = 19.27, *p* = 0.0001) and the interaction between age and genotype (F(1,30) = 5.657, *p* = 0.024) were significant, whereas genotype alone was not, indicating a more complex, age-modulated effect of disease in this region.

In addition, significant age-related cholesterol accumulation, measured as the percentage of Filipin+ area, was detected in the cervical and thoracic white matter of the spinal cord in both WT and 5xFAD mice at 9 months compared with 3 months of age (Figure 7C,D). Two-way ANOVA revealed a strong main effect of age in all spinal cord regions (cervical: F(1,28) = 24.29, *p* < 0.0001; thoracic: F(1,28) = 19.96, *p* = 0.0001; lumbar: F(1,28) = 14.78, *p* = 0.001), whereas no significant main effects of genotype or age × genotype interaction were observed in any region. Consistent with this, age-related increases in the Filipin+ area were observed in the cervical and thoracic white matter in both WT and 5xFAD mice, indicating a generalized effect of aging rather than disease specificity. In the lumbar spinal cord white matter, 9-month-old 5xFAD mice exhibited significantly greater cholesterol accumulation compared with 3-month-old 5xFAD mice, whereas no significant age-related difference was detected in WT mice (Figure 7C,D). These findings suggest that, in the white matter of the spinal cord, Iba+ microglial cell-mediated neuroinflammation is driven by both ageing and AD pathology, whereas lipid dysregulation appears to be primarily age-related and not specific to AD.

### 2.8. Sex Differences in Spinal Cord Pathology in 5xFAD Mice

Sex-specific differences in AD incidence have been reported in patients. However, the cellular and molecular mechanisms underlying sex-dependent CNS pathology in AD remain poorly characterized. To investigate this, we evaluated key pathological features in the spinal cord of female and male 5xFAD and WT mice, including amyloid deposition, neuronal loss, and glial cell-mediated neuroinflammatory responses.

**Figure 8:**
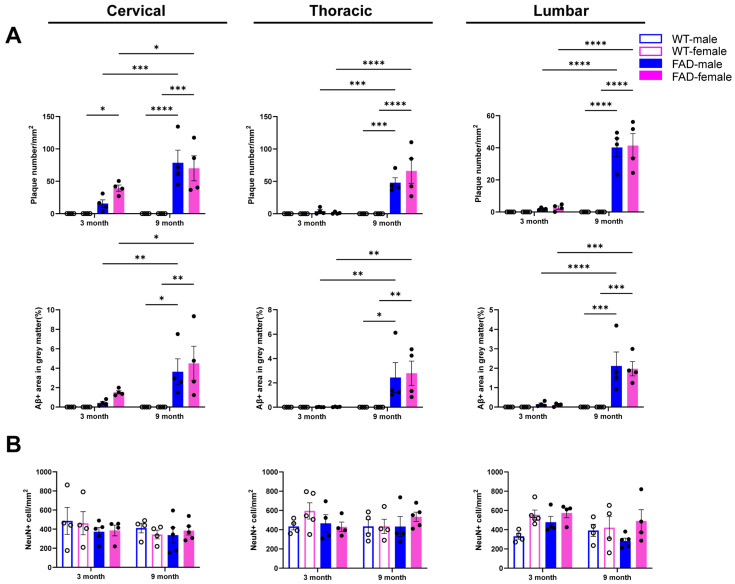
Sex differences in Aβ accumulation and neuronal loss in the AD spinal cord. Data from female and male mice were stratified by sex and reanalysed for comparison. (**A**) Quantification of the percentage of Aβ-covered area and plaque number in the grey matter of the cervical, thoracic and lumbar spinal cord in female and male WT and 5xFAD mice. (**B**) Quantification of NeuN+ neuronal cell density in the ventral horn of the cervical, thoracic and lumbar spinal cord in female and male WT and 5xFAD mice. Data are presented as the mean ± SEM (*n* = 4–5 mice per group). *: *p* < 0.05, **: *p* < 0.01, ***: *p* < 0.001, and ****: *p* < 0.0001.

After stratifying the data by sex, we found that both 9-month-old female and male 5xFAD mice exhibited significantly increased Aβ deposition across all three spinal cord regions compared with WT controls and 3-month-old 5xFAD mice (Figure 8A). Consistent with the mixed-sex analysis (Figure 2), no significant differences in NeuN+ cell counts were observed in the ventral cervical, thoracic, or lumbar spinal cord between WT and 5xFAD mice in either sex (Figure 8B). Furthermore, no significant differences were observed between female and male 5xFAD mice in Aβ-covered area, plaque density, or neuronal cell density at either 3 or 9 months of age (Figure 8A,B).

A three-way ANOVA (age × genotype × gender) revealed consistent effects of age and genotype on Aβ burden and plaque number across all spinal cord regions. In contrast, no significant main effects of gender were detected, and no significant interactions involving gender were observed, indicating that sex does not significantly influence Aβ pathology or plaque burden in this model.

**Figure 9:**
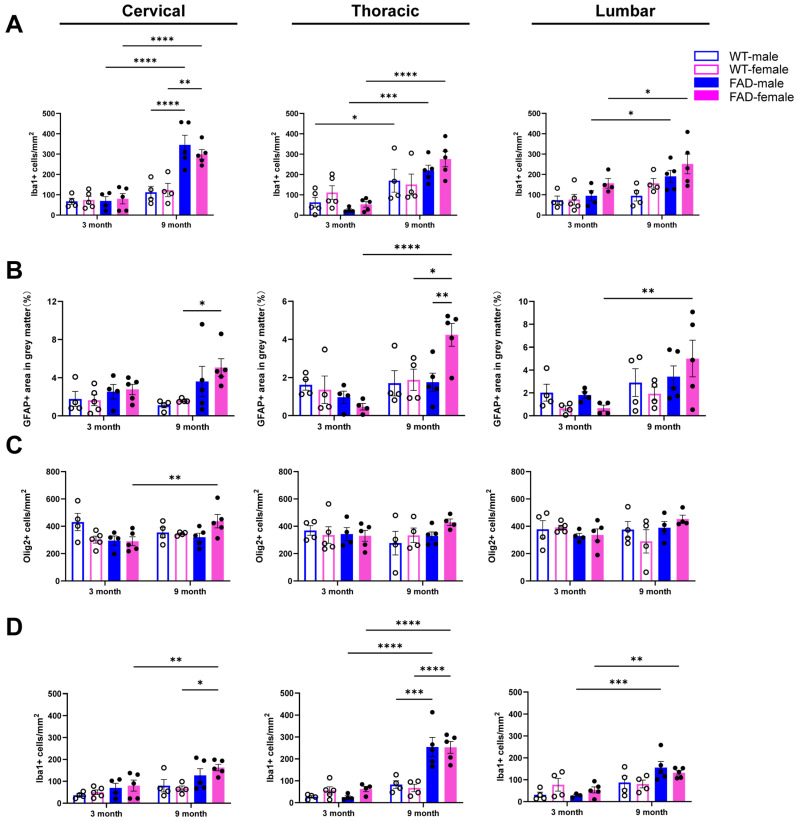
Sex-specific glial responses in the spinal cord of WT and 5xFAD mice. Data from female and male mice were stratified by sex and reanalysed for comparison. (**A**–**C**) Quantification of Iba1+ cell density (**A**), percentage of GFAP+ area (**B**), and Oligo2+ cell density (**C**) in the grey matter of the cervical, thoracic and lumbar spinal cord in female and male WT and 5xFAD mice. (**D**) Quantification of Iba1+ cell density in the white matter of the cervical, thoracic and lumbar spinal cord of female and male WT and 5xFAD mice. Data are presented as the mean ± SEM (*n* = 4–5 mice per group). *: *p* < 0.05, **: *p* < 0.01, ***: *p* < 0.001, and ****: *p* < 0.0001.

Microglial density (Iba1+ cells) was shown to be significantly increased in the grey matter of various spinal cord regions of 9-month-old mice compared to 3-month-old mice in both female and male 5xFAD mice. However no significant difference was observed between age- and genotype-matching female and male groups (Figure 9A,D). A three-way ANOVA showed strong age effects on Iba1 immunoreactivity across all spinal cord regions. In the cervical region, significant effects of age (F(1,28) = 50.32, *p* < 0.0001), genotype (F(1,28) = 23.38, *p* < 0.0001), and an age × genotype interaction (F(1,28) = 8.06, *p* = 0.008) were observed. In the thoracic and lumbar regions, significant age effects were also detected (thoracic: F(1,29) = 34.69; lumbar: F(1,27) = 26.35; both *p* < 0.0001), along with significant age × genotype interactions (thoracic: F(1,29) = 8.06, *p* = 0.008; lumbar: F(1,27) = 7.44, *p* = 0.011). Genotype and sex effects were mostly non-significant, and no significant sex-related effects were detected, indicating that microglial changes are primarily age-driven and modulated by genotype. In contrast, astrocytic reactivity, measured as the percentage of GFAP+ area, was significantly higher in the thoracic spinal cord of 9-month-old female 5xFAD mice compared with age-matched males (Figure 9B). Compared with WT littermates, 9-month-old female 5xFAD mice also exhibited significantly increased GFAP coverage in the cervical and thoracic regions, whereas age-matched male 5xFAD mice showed no significant differences relative to their WT counterparts. Furthermore, an age-dependent increase in GFAP+ area (9 months vs. 3 months) in the thoracic and lumbar spinal cord was observed in female, but not male, 5xFAD mice. Three-way ANOVA analysis indicates that astrocytic GFAP+ area showed region- and sex-specific changes in 5xFAD mice. In the cervical spinal cord, a significant main effect of genotype was observed (F(1,28) = 10.10, *p* = 0.004), with no effects of age, gender, or interactions. In the thoracic region, significant effects of age (F(1,26) = 12.37, *p* = 0.002) and age × genotype (F(1,26) = 7.257, *p* = 0.012) and age × gender (F(1,26) = 5.421, *p* = 0.028) were detected. In the lumbar spinal cord, only age showed a significant effect (F(1,26) = 9.015, *p* = 0.006). Overall, the increase in GFAP coverage was driven by age, with stronger and interaction-dependent effects in females. 

**Figure 10:**
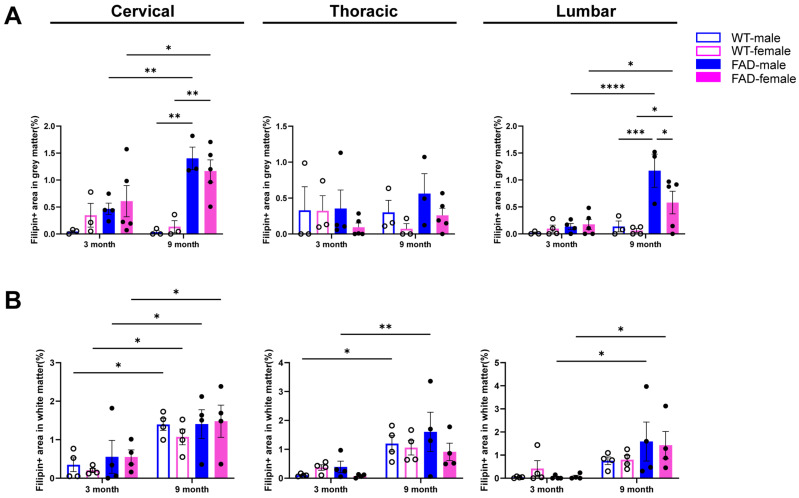
Sex-specific differences in free cholesterol accumulation in the spinal cord of 5xFAD mice. Data from female and male mice were stratified by sex and reanalysed for comparison. Quantification of Filipin+ area in the grey (**A**) and white matter (**B**) of the cervical, thoracic and lumbar spinal cord in female and male WT and 5xFAD mice. Data are presented as the mean ± SEM (*n* = 3–5 mice per group). *: *p* < 0.05; **: *p* < 0.01; ***: *p* < 0.001; and ****: *p* < 0.0001.

For free cholesterol levels, both female and male 9-month-old 5xFAD mice showed a significant increase in Filipin+ staining in the grey matter of the cervical and lumbar spinal cord compared to 3-month-old mice or the age-matched WT controls. Notably, in the lumbar spinal cord of 9-month-old 5xFAD mice, males showed significantly higher Filipin+ signals than age-matched females. Furthermore, the age-dependent increase in Filipin+ staining in the white matter of the thoracic spinal cord was observed only in male WT and 5xFAD mice (Figure 10A,B). Three-way ANOVA analysis suggests that Filipin+ staining was region-specific and sex-dependent changes in 5xFAD mice. No consistent gender effects or interactions were detected, indicating predominantly age- and genotype-driven cholesterol accumulation. 

These findings suggest that astrocytic responses in the spinal cord were more pronounced in female 5xFAD mice, whereas cholesterol accumulation was found to be higher in certain spinal regions of male mice. In contrast, amyloid deposition and microglial activation appeared largely sex independent.

## 3. Discussion

In this study, we systematically examined spinal cord pathology in 5xFAD mice at the age of 3 and 9 months, with a focus on amyloid deposition, neuronal loss, neuroinflammation, cholesterol metabolism, and potential sex-specific differences. While previous studies have reported a higher prevalence of AD in elderly females and the influence of sex hormones on neuroinflammation, spinal cord pathology has largely been studied in a single sex, or the sex of the subjects was not specified in the study [21,22,23]. Our findings provide new insights into the interactions between glial and neuronal cells and amyloid pathology in both male and female mice, revealing important age- and sex-related differences.

We observed early accumulation of Aβ plaques in the cervical spinal cord of 3-month-old 5xFAD mice, with a subsequent spread to thoracic and lumbar regions by 9 months. Plaques were predominantly localized to grey matter, with dorsal lamina largely spared. These findings are consistent with previous reports [18,22] and highlight regional differences in amyloid deposition in the CNS. The ventral localization of Aβ aligns with the predominance of motor neurons in these laminae [15] and may therefore contribute to motor dysfunction in AD. Although motor and sensory functions were not assessed in our study, previous reports have demonstrated age-dependent deficits in motor performance and coordination in 5xFAD mice, evident at 12 months of age but not at 3 months (18). Unlike TgCRND8 mice, where spinal Aβ partially originates from corticospinal projections [24], our results suggest local Aβ accumulation in the 5xFAD spinal cord. While Aβ deposition initiates concurrently in the brain and spinal cord, its progression is slower in the cervical spinal cord when compared with the hippocampus and cortex, suggesting differences between the brain and spinal cord in deposition or clearance mechanisms [20,25]. Despite early plaque deposition, no significant neuronal loss was detected in the ventral spinal cord at 3 or 9 months. This is consistent with previous reports showing that the number of cervical motor neurons in 5xFAD mice is comparable to WT littermates at 6 months [21]. However, the lack of overt neuronal loss does not exclude the possibility of underlying neuronal dysfunction, synaptic alterations, or early degenerative changes that may precede cell loss and contribute to functional impairment. A significant reduction in NeuN+ cells was observed only at 12 months and was restricted to the cervical and lumber spinal cord compared with WT controls [22]. This finding supports the idea that spinal cord neurons are relatively resilient to early Aβ accumulation, similar to observations in the lumbar spinal cord of Tg2576 mice, where neuron loss occurs at later stages (10 months) [23].

Microglial reactivity, as assessed by Iba1 staining, was minimal in the spinal cord tissues at 3 months but significantly increased at 9 months in 5xFAD mice, suggesting a combined effect of disease progression and aging. However, it is important to note that Iba1 primarily reflects microglial presence rather than functional phenotype and hence the effect of this increase on activation state remains to be established. Astrocytic immunoreactivity has previously been reported to increase in the spinal cord of 12-month-old 5xFAD mice [22]. In our study, astrocytic reactivity, qualified by GFAP+ staining area, was already significantly elevated at 9 months in the cervical and thoracic regions. Consistent with age-dependent glial changes, an increased number of oligodendrocytes has been reported in the cortex and corpus callosum of 10-month-old, but not 4-month-old, 5xFAD mice [26]. Similarly, our data show that the number of Olig2^+^ oligodendrocytes in the cervical spinal cord increases with age; however, this effect was observed only in female 5xFAD mice. Correlation analysis further revealed that the levels of Aβ burden were positively correlated with glial cell activation, especially in thoracic and lumber regions. The weaker correlation in the cervical region possibly suggests that additional factors may contribute to glial activation beyond Aβ. Collectively, these results suggest that glial cell responses in the grey matter of the spinal cord of 5xFAD mice occur primarily at later stages of the disease. Despite early Aβ accumulation in the cervical spinal cord at 3 months, microglial, astrocytic, and oligodendrocyte responses were not evident until 9 months of age.

AD is a neurodegenerative disease reported to be more prevalent in females [27]. In our study, while Aβ deposition, neuronal density, and microglial activation were largely sex-independent, astrocytic reactivity was significantly more pronounced in female 5xFAD mice at 9 months. Females also exhibited stronger correlations between Aβ deposition and GFAP expression, highlighting sex-specific vulnerability of astrocytes in AD development. These observations are consistent with previous reports linking astrocytes to sex differences in neuroinflammation in aging [28]. Female-biased astrocytic priming has also been shown to shape early locus coeruleus vulnerability in the presence of Aβ oligomers [29]. Additionally, increases in hippocampal GFAP immunoreactivity and GFAP+ cell counts following ethanol exposure have been reported to be more robust in females than in males [30]. Together, our results from spinal cord tissue analysis in 5xFAD mice suggest that astrocytic responses in AD may exhibit sex-specific differences. In particular, we observed a sex-biased pattern of astrocytic activation in the spinal cord of this model. While these findings point to a potential role for sex-dependent astrocytic responses, further studies across multiple regions and time points are required to determine their relevance to the higher prevalence and differential progression of AD in females.

Filipin staining showed no difference in spinal cord cholesterol levels between WT and 5xFAD mice at 3 months, despite early Aβ deposition. In contrast, at 9 months, 5xFAD mice exhibited significant cholesterol accumulation in the grey matter of the cervical and lumbar spinal cord, coinciding with enhanced neuroinflammatory responses. This was accompanied by reduced mRNA expression of *Cyp46a1*, *Cyp27a1*, and *Abca1*, consistent with impaired cholesterol transport and clearance and providing a mechanistic basis for lipid accumulation [31]. Notably, increased Filipin positivity in the white matter was age-dependent and independent of amyloid pathology. Together, these findings indicate that lipid dysregulation in the spinal cord emerges after amyloid deposition and parallels the delayed onset of glial activation. Cholesterol dysregulation has been implicated in promoting amyloidosis, chronic inflammation, myelin disruption, and blood–brain barrier impairment, highlighting the complex interplay between lipid metabolism and neurodegeneration [13]. In aging and neurodegenerative diseases, the accumulation of lipid droplets, likely induced by excess free cholesterol, has been observed in microglia [32] and is associated with a dysfunctional and proinflammatory state [33]. Moreover, lipid droplet accumulation has been reported to impair microglial phagocytic capacity, thereby reducing Aβ clearance [34].

While inflammation and amyloid burden in the grey matter are directly linked to AD pathogenesis, white matter abnormalities, such as microglial activation and focal lesions, have been associated with an increased risk of stroke [35] and dementia [20]. Our findings support previous reports in aged and AD brains [36], demonstrating that despite a lower amyloid burden than in grey matter, white matter in 9-month-old 5xFAD mice exhibited elevated microglial activation compared with WT controls. Furthermore, while the observed correlation between Aβ deposition and neuroinflammatory markers suggests an association, Aβ appears to account for only part of the variability, and therefore these results should be interpreted with appropriate caution. Additionally, age-dependent increases in cholesterol accumulation were observed in both WT and 5xFAD mice, indicating that lipid changes in white matter may occur independently of Aβ pathology.

In summary, this study demonstrates that Aβ accumulation occurs early in the cervical spinal cord of 5xFAD mice, whereas glial activation and cholesterol accumulation emerge later, at 9 months. Astrocytic activation exhibits a female-specific predominance, highlighting sex-based differences in spinal cord pathology in AD. These findings advance our understanding of spinal cord involvement in AD, providing a mechanistic link to the motor dysfunction that develops after cognitive decline. By characterizing age- and sex-dependent changes, this study highlights the importance of considering spinal cord pathology and sex differences in AD research and therapeutic development.

## 4. Materials and Methods

### 4.1. Animals

Wild-type and heterozygous 5xFAD mice on a C57BL/6J background (B6.Cg-Tg(APPSwFlLon, PSEN1M146LL286V)6799Vas/Mmjax) were bred and maintained in the Biological Procedures Unit at the University of Strathclyde. Female and male 5xFAD mice and their WT littermates at 3 and 9 months of age were used in this study. The number of animals per group is stated in the legend of each figure. All procedures were performed in accordance with the UK Animals (Scientific Procedures) Act 1986, under a UK Home Office project licence (PPL6183228), and were approved by the University of Strathclyde Animal Welfare and Ethical Review Body.

Genotyping was conducted following the recommended protocols and primer sequences provided by The Jackson Laboratory. Genomic DNA was extracted from ear biopsies and amplified using the Phire Tissue Direct Master Mix (Thermo Fisher Scientific, Paisley, UK). PCR products were separated by agarose gel electrophoresis in TAE buffer. Samples exhibiting both the WT band (216 bp) and the transgene band (129 bp) were identified as heterozygous 5xFAD mice, whereas samples showing only the WT band were identified as WT animals.

### 4.2. Tissue Collection and Processing

Mice were euthanised in a CO_2_-filled chamber and transcardially perfused with 30 mL of phosphate-buffered saline (PBS). The spinal cord was extruded by flushing the vertebral canal with sterile PBS using a 19-gauge needle and syringe. The spinal cord was then subdivided into cervical, thoracic, and lumbar segments according to established anatomical landmarks, including the cervical and lumbar enlargements.

For immunofluorescence analysis, tissues were embedded in optimal cutting temperature (OCT) compound (VWR Chemicals, Lutterworth, UK), snap-frozen on dry ice, and sectioned at 10 μm using a Leica CM1950 cryostat (Leica Microsystems (UK) Ltd., Sheffield, UK). Sections were air-dried, wrapped in aluminium foil, and stored at −20 °C until further processing.

### 4.3. Fluorescence Immunohistochemistry

Spinal cord sections were fixed either in ice-cold 25% acetone/75% ethanol or in 10% neutral buffered formalin for 15 min, according to antibody-specific requirements. For formalin-fixed tissues, heat-induced antigen retrieval (HIER) was performed by microwaving slides in antigen retrieval buffer at the low power setting for 10 min after the solution reached boiling.

Following two washes with Tris-buffered saline (TBS), a hydrophobic barrier was drawn around each section using ImmEdge Pen (Vector Laboratories, Newark, CA, USA). Sections were rehydrated in TBS and incubated in blocking buffer (3%BSA in TBS with 0.05% Tween 20 and 0.1% Triton-X) for 1 h. Following this, primary antibodies were applied overnight at 4 °C in blocking buffer at pre-determined optimal concentrations. The antibodies purchased from Cell Signalling Technology Europe B.V. (London, UK) include: rabbit anti-β amyloid (8243), mouse anti-βamyloid (15126), rabbit anti–Iba-1 monoclonal antibody (019-19741), and mouse anti-GFAP monoclonal antibody (3670). The other antibodies used include: rabbit anti-Olig2 polyclonal antibody (MABN50, Merck millipore, Mumbai, India) and guinea-pig anti-NeuN antibody (ABN90, Merck (Sigma-Aldrich), Gillingham, UK). Slides were then washed three times in TBST (TBS with 0.05% Tween-20) for 5 min each and incubated with secondary antibodies, including donkey anti-rabbit IgG(H+L) Alexa Flour TM488 (A21206, Invitrogen, Paisley, UK), donkey anti-rabbit IgG(H+L) Alexa Flour TM647 (A31573, Invitrogen) goat anti-guinea-pig IgG(H+L) Alexa Flour 488 (A11073, Invitrogen) and donkey anti-mouse IgG(H+L) Alexa Fluor 555 (ab150110, Abcam, Cambridge, UK). After three additional TBST washes (5 min each), nuclei were counterstained with DAPI (Invitrogen), and sections were mounted with ProLong Gold Antifade Mountant (Invitrogen).

For Filipin staining, sections were fixed in 4% paraformaldehyde for 15 min, washed twice in PBS, and incubated with Filipin III (25 μg/mL; Merck (Sigma-Aldrich), Gillingham, UK) for 1 h at room temperature before mounting with ProLong Gold Antifade Mountant.

Images were acquired using a Leica SP8 confocal microscope (Leica Microsystems (UK) Ltd., Sheffield, UK. Representative images are shown as maximum-intensity projections of three optical sections collected at 4 μm intervals.

### 4.4. Data Processing and Immunofluorescence Image Analysis

Representative images were selected from animals with values closest to the group mean. Image processing was performed using ImageJ 1.52, and quantitative analyses were conducted using QuPath 0.5.0. Coverage thresholds were applied for area-based measurements, and positive cell quantification was performed using QuPath’s cell detection and trained object classifier functions. Aβ burden in the grey matter was quantified by the percentage area covered by staining and the number of plaques per mm^2^, with plaques ≥ 100 μm^2^ included in the analysis to exclude very small, non-specific signals and potential staining artefacts, thereby minimising histological noise and ensuring robust quantification of plaques. GFAP immunoreactivity was quantified as the percentage area of GFAP-positive staining in the grey matter. Expression of Iba1, Olig2, and NeuN was quantified as the number of positive cells per mm^2^. Only cells colocalising with DAPI were included in the analysis, and signals not overlapping with DAPI were excluded. A minimum of three sections per animal were stained and analysed, and quantification was performed on sections meeting predefined quality criteria (signal-to-noise ratio and structural integrity) to ensure consistency across samples. The measurements were averaged per animal before statistical analysis.

### 4.5. RNA Extraction, cDNA Synthesis and Real Time-Quantitative PCR (RT-qPCR)

RNA was extracted from cervical, thoracic, and lumbar spinal cord tissues. Samples were homogenized in 300 µL of TRIzol Reagent (Life technology, Carlsbad, CA, USA), followed by protein denaturation using 200 µL of chloroform (Merck (Sigma-Aldrich), Gillingham, UK) twice. RNA was precipitated with 300 ul of 2-propanol containing 30 µL of 5M sodium acetate and 1ul of glycogen. The resulting RNA pellet was washed three times with 75% RNase free ethanol and subsequently dissolved in 30 ul of RNase-free water. RNA concentration and purity were assessed using a NanoDrop 2000C spectrophotometer (Thermo Fisher Scientific). cDNA synthesis was performed using the QuantiTect Reverse Transcription Kit (Qiagen, Hilden, Germany) according to the manufacturer’s instructions. RT–qPCR was conducted to assess gene expression levels. All samples were analysed in technical triplicates using the PowerUp SYBR Green PCR Master Mix (Applied Biosystems, Thermo Fisher Scientific, Paisley, UK). Primer sequences used for amplification included *Gapdh* (Forward: CCCACTAACATCAAATGGGG; Reverse: CCTTCCACAATGCCAAAGTT), *Abca1* (Forward: CCCACTAACATCAAATGGGG; Reverse: CCTTCCACAATGCCAAAGTT), *Abcg1* (Forward: ACCTACCACAACCCAGCAGACTTT; Reverse: GGTGCCAAAGAAACGGGTTCACAT), *Cyp27a1* (Forward: GCCTTGCACAAGGAAGTGACT; Reverse: CGCAGGGTCTCCTTAATCACA), *Cyp46a1* (Forward: CCCTAGCCTTTCCCCAAATTGC; Reverse: CGGAAGAATCCCTTGCAACC), and *Lxrα* (Forward: CCCTAGCCTTTCCCCAAATTGC; Reverse: CGGAAGAATCCCTTGCAACC). Cycle threshold (Ct) values were normalized to Gapdh and calibrated to WT control samples. Relative gene expression levels were calculated using the 2^−ΔΔCt^ method.

### 4.6. Statistical Analysis

Data are presented as the mean ± standard error of the mean (SEM), unless stated otherwise. Statistical analyses were performed using GraphPad Prism 10. Comparisons between groups were conducted using one-way, two-way or three-way ANOVA, followed by Tukey’s post hoc test for multiple comparisons. A *p*-value < 0.05 was considered statistically significant.

## Data Availability

Data generated are available at https://doi.org/10.15129/e2e03e27-29eb-4df0-b3ef-e7a4cb7ee368.

## Abbreviations

The following abbreviations are used in this manuscript:

AD	Alzheimer’s disease
WT	Wild type
Aβ	β-amyloid
CNS	Central nervous system
GM	Grey matter
WM	White matter
APP	Amyloid precursor protein
OCT	Optimal cutting temperature
ANOVA	Analysis of variance
PCR	Polymerase chain reaction
RNA	Ribonucleic acid
DNA	Deoxyribonucleic acid
PBS	Phosphate-buffered saline
DAPI	4′,6-diamidino-2-phenylindole
TBS	Tris-buffered saline
TAE	Tris-acetate-EDTA
